# Limb Salvage in a War Zone: Reconstruction of Extensive Radial Bone Loss Using a Nonvascularized Fibular Graft and Groin Flap

**DOI:** 10.7759/cureus.112208

**Published:** 2026-07-07

**Authors:** Laith Hseinat, Ahmad Almigdad, Majdala Al-Bataineh, Mutaz M Alnaser, Mohammed Abualfoul, Marwan Abo Hashim, Mohammad Altheabat, Malek Alrbehat, Hidar Al Soudi

**Affiliations:** 1 Department of Orthopedics, Jordanian Royal Medical Services, Amman, JOR; 2 Faculty of Medicine, Yarmouk University, Irbid, JOR; 3 Department of Plastic and Reconstructive Surgery, Jordanian Royal Medical Services, Amman, JOR; 4 Department of Orthopedics, Ministry of Health - Jordan, Amman, JOR

**Keywords:** blast injury, fibular graft, forearm reconstruction, groin flap, limb salvage, radial bone loss, radius nonunion, segmental bone loss

## Abstract

High-energy blast injuries frequently result in complex bone and soft-tissue defects that pose significant reconstructive challenges, particularly in conflict zones where access to specialized surgical care is limited. Delayed treatment, infection, and soft-tissue compromise further complicate management and may lead to severe functional impairment. Segmental radial bone loss associated with extensive soft-tissue injury is especially difficult to reconstruct, often requiring staged procedures to achieve limb salvage and functional restoration. We report the case of a 17-year-old female who sustained a blast injury to her left forearm in Gaza, resulting in extensive soft-tissue destruction, segmental radial bone loss, and subsequent infection. Initial management was constrained by the collapse of local health care services during the conflict, necessitating evacuation from northern to southern Gaza to access specialized reconstructive care. Following serial debridement and successful infection control, reconstruction was performed using a nonvascularized fibular graft to address the radial defect, combined with a groin flap for soft-tissue coverage. The patient underwent staged surgical management, achieving successful graft incorporation, complete wound healing, restoration of limb stability, and satisfactory functional recovery.

## Introduction

The protracted conflict in Gaza has led to widespread destruction of civilian infrastructure, substantial loss of life, and a severe humanitarian crisis. Ongoing hostilities have generated a considerable burden of complex traumatic injuries while simultaneously disrupting health care delivery through damage to medical facilities, shortages of essential supplies, and limited access to specialized surgical care [1]. In a recent analysis of 362 orthopedic patients treated during the conflict, blast injuries and gunshot wounds accounted for the majority of cases, with complex extremity fractures and extensive soft-tissue injuries representing a significant proportion of the orthopedic workload. These conditions frequently result in delayed definitive treatment and increase the risk of infection, nonunion, permanent disability, and preventable mortality [2,3].

Blast injuries to the extremities often cause extensive destruction of bone and soft tissues, including muscles, tendons, nerves, and vascular structures. Segmental bone loss associated with severe soft-tissue compromise represents one of the most challenging reconstructive problems in orthopedic trauma, frequently requiring staged procedures, complex soft-tissue coverage, and prolonged rehabilitation. In resource-constrained and conflict settings, these challenges are further compounded by the limited availability of advanced reconstructive techniques and delayed access to definitive care [4].

We report the case of a 17-year-old female who sustained a Gustilo-Anderson type IIIB open fracture of the left forearm following a blast injury in northern Gaza. Her injury was complicated by infection, hardware exposure, extensive soft-tissue loss, and a large segmental radial bone defect. Following serial debridement and infection control, limb salvage was successfully achieved using a nonvascularized fibular graft (NVFG) for reconstruction of the radial defect, combined with a groin flap for soft-tissue coverage, resulting in successful bone union and satisfactory functional recovery.

## Case presentation

**Figure 1:**
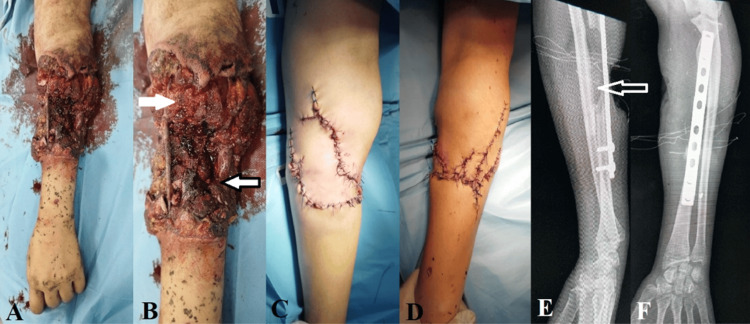
Initial injury and primary surgical management (A, B) Initial injury photographs demonstrating the severity of the injury, with extensive soft-tissue destruction and severe contamination. (C, D) Intraoperative photographs from the primary surgery showing soft-tissue debridement and initial wound closure with radius fixation using a plate. (E, F) Postoperative anteroposterior (F) and lateral (E) radiographs demonstrating the radial bone defect.

**Figure 2:**
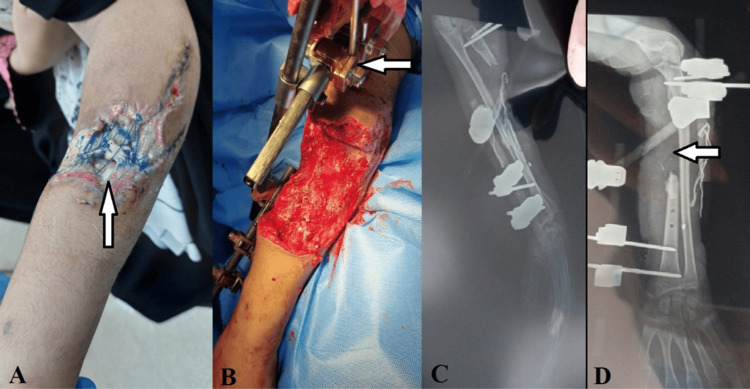
Skin necrosis, repeat debridement, and external fixation (A) Two-week follow-up clinical photograph demonstrating the extent of skin necrosis and the soft-tissue defect. (B) Intraoperative photograph following the second debridement, with removal of the plate and application of an external fixator, showing the extent of soft-tissue loss. (C, D) Postoperative anteroposterior (D) and lateral (C) radiographs demonstrating external fixation and the bone defect following plate removal.

A 17-year-old female sustained a blast injury in May 2024 in the northern Gaza Strip, resulting in a severe left forearm injury characterized by an open fracture of the radius with segmental bone loss (Gustilo-Anderson type IIIB) (Figure 1). Initial management consisted of multiple surgical debridements followed by open reduction and internal fixation with a dynamic compression plate and soft-tissue coverage using a skin flap. Subsequently, the patient developed a deep infection complicated by flap necrosis, leading to exposure of the fixation hardware and a persistent soft-tissue defect (Figure 2). She therefore underwent implant removal, repeated debridement procedures, and stabilization of the radius using an external fixator.

The patient sought treatment at multiple health care facilities in northern Gaza. However, owing to the severity and complexity of the injury, combined with limited local resources, including the lack of specialized upper-limb reconstructive and plastic surgery expertise, upper-limb amputation, either above or below the elbow, was recommended as the primary treatment option.

**Figure 3:**
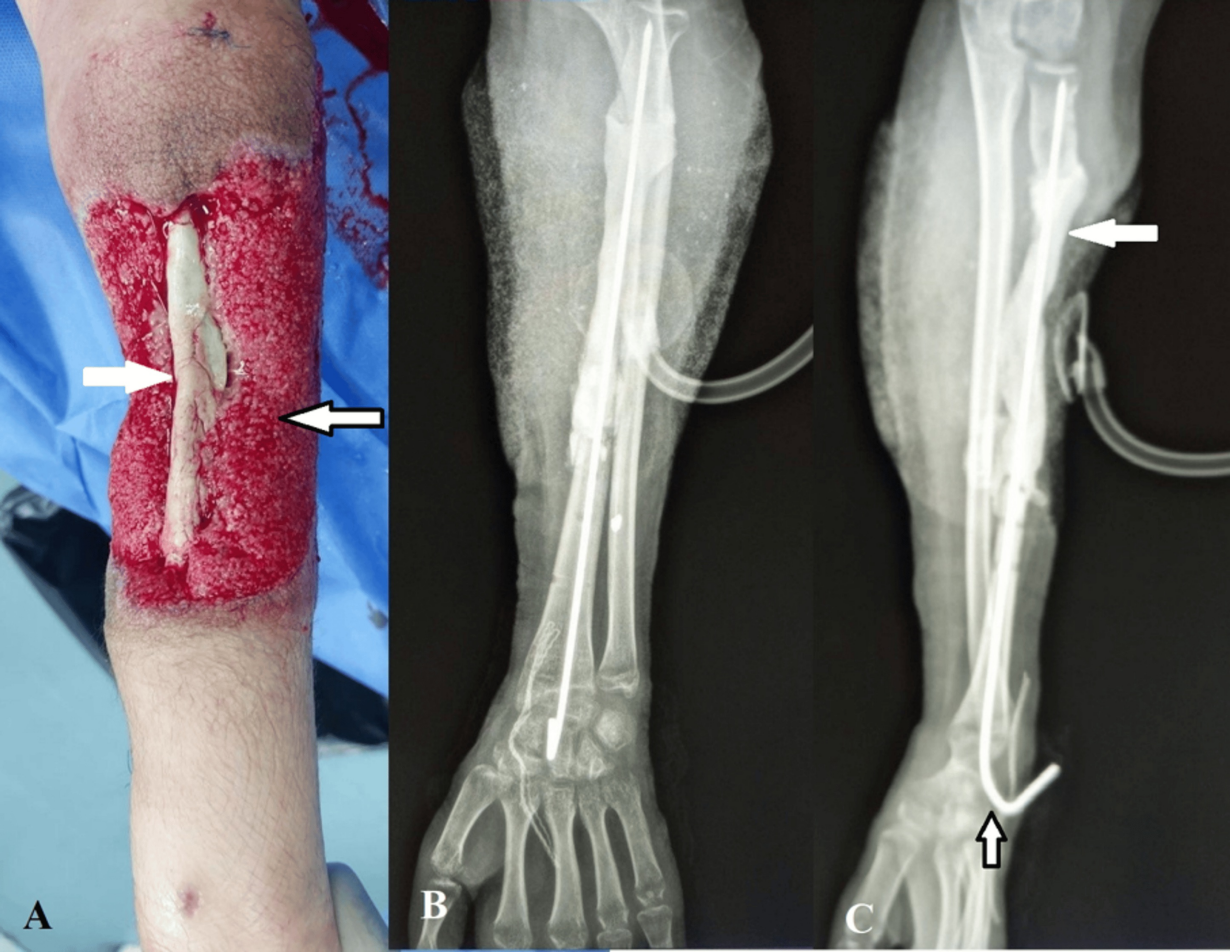
Granulation tissue formation following VAC therapy and bone cement application (A) Intraoperative photograph demonstrating healthy granulation tissue within the soft-tissue defect following application of VAC therapy and bone cement, used to fill the osseous gap between the proximal and distal bone ends and around the intramedullary fixation to promote induced membrane formation and aid infection control. (B, C) Postoperative anteroposterior (B) and lateral (C) radiographs showing intramedullary fixation with bone cement occupying the bone defect. VAC, vacuum-assisted closure

Following multidisciplinary evaluation and consultation with the Jordanian Field Hospital in the northern Gaza Strip, a limb-salvage strategy was deemed feasible despite the anticipated need for prolonged treatment, multiple surgical procedures, and intensive follow-up. The patient subsequently underwent staged limb reconstruction, including serial surgical debridements, placement of an antibiotic-loaded bone cement spacer for infection control and defect management, intramedullary stabilization, and vacuum-assisted closure (VAC) therapy (Figure 3).

Nevertheless, definitive reconstruction required the transfer of the patient from northern Gaza to southern Gaza. Owing to the ongoing conflict and restrictions on movement, medical evacuation proved challenging and required extensive coordination with the World Health Organization. After approximately three months, a humanitarian medical evacuation was successfully arranged, allowing the patient to proceed with definitive treatment.

**Figure 4:**
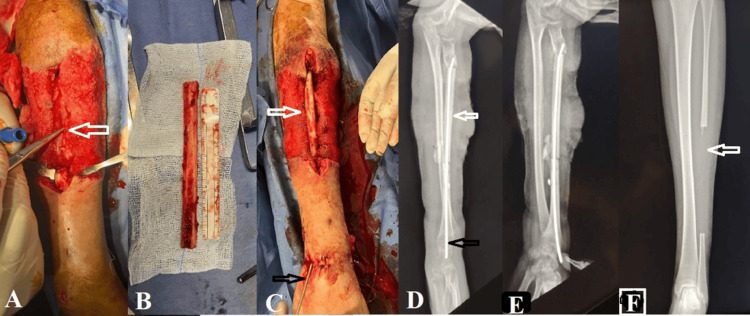
Second-stage reconstruction with NVFG and intramedullary fixation (A) Intraoperative photograph demonstrating healthy soft-tissue conditions with well-formed granulation tissue and a well-defined induced membrane following removal of the cement spacer. (B) Photograph showing the harvested NVFG in its full length. (C) Intraoperative photograph illustrating the final positioning of the fibular graft following fixation with elastic stable intramedullary nails. (D, E) Three-week postoperative follow-up radiographs in lateral (D) and anteroposterior (E) views demonstrating satisfactory alignment and positioning of the fibular graft with elastic nail fixation. (F) Postoperative anteroposterior radiograph showing the fibular donor site. NVFG, nonvascularized fibular graft

**Figure 5:**
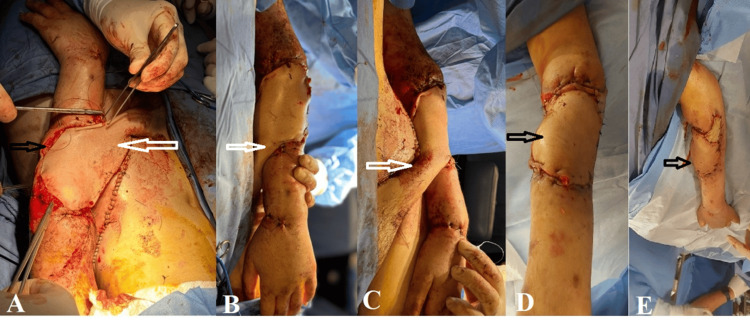
Groin pedicled flap reconstruction and flap division (A-C) Intraoperative photographs demonstrating the groin pedicled flap and final positioning of the forearm with complete coverage of the soft-tissue defect during the first-stage flap procedure. (D, E) Intraoperative photographs showing the final appearance of the forearm following the second-stage procedure, including flap division and inset, completing soft-tissue reconstruction.

Following evacuation, the patient underwent additional debridement sessions and VAC therapy until optimization for definitive reconstruction was achieved. Revision surgery involved reconstruction of a 12-cm radial defect using a 12.5-cm NVFG. Fixation was performed with an elastic stable intramedullary nail (Figure 4). During the same procedure, the forearm was temporarily implanted into the groin wall to achieve full-thickness soft-tissue coverage for three weeks (Figure 5). Subsequently, the limb was detached in a second-stage procedure, and the groin wall defect was closed primarily. The patient remained hospitalized for an additional week for wound care and close monitoring.

**Figure 6:**
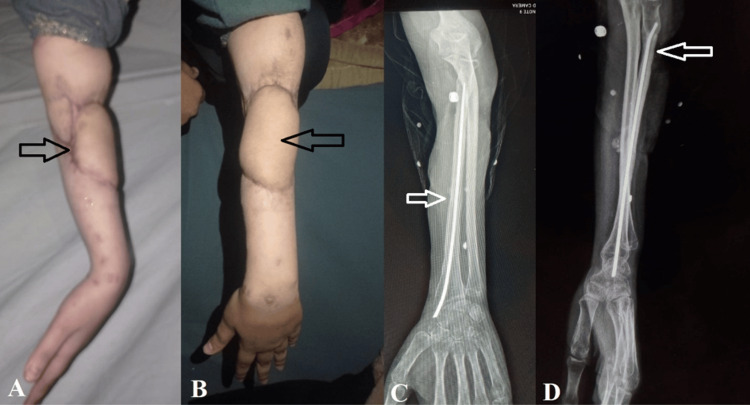
Final clinical outcome and radiographic bone healing (A, B) Four-month follow-up clinical photographs demonstrating complete soft-tissue healing and the final appearance of the affected forearm. (C, D) Six-month follow-up radiographs in anteroposterior (C) and lateral (D) views demonstrating complete graft incorporation with satisfactory bone healing and maintained alignment.

The patient was subsequently followed up regularly; however, due to difficulties accessing the hospital, follow-up was conducted via telephone consultations supported by clinical updates and radiologic assessments. No evidence of infection was observed, and satisfactory graft incorporation with progressive bone healing was achieved within four months (Figure 6). The last in-person follow-up at our hospital was at 18 months, at which time the patient demonstrated good functional recovery; however, she had persistent wrist drop, for which tendon transfer was indicated.

## Discussion

Blast injuries result from high-energy forces, causing simultaneous soft-tissue, vascular, and skeletal destruction, often leading to complex open fractures, segmental bone loss, and extensive soft-tissue compromise. These injuries are frequently associated with nerve and tendon damage and carry a high risk of long-term functional impairment. Effective management requires staged reconstruction with meticulous attention to both osseous stabilization and soft-tissue coverage [5].

In conflict settings, timely management is further compromised by overwhelmed health care systems, limited resources, and delayed access to specialized care. These factors increase the risk of infection, tissue necrosis, and ultimately limb loss, making early multidisciplinary intervention essential for limb salvage [6].

Segmental bone defects exceeding the critical size require structural reconstruction. Although vascularized fibular grafts are widely regarded as the gold standard because of their intrinsic blood supply and enhanced biological potential, their application is limited by the need for microsurgical expertise, longer operative times, and advanced perioperative infrastructure, which may not be available in low-resource or conflict settings [7].

NVFGs remain a practical and effective alternative in appropriately selected cases, particularly when infection is adequately controlled and stable fixation is achieved. Reported union rates for NVFGs in the literature vary widely but can approach those of vascularized techniques in selected patients with favorable local conditions [8]. In the present case, meticulous infection control through serial debridement, VAC, and antibiotic-loaded cement spacer placement likely contributed to the development of a biologically active induced membrane, thereby enhancing graft incorporation in a manner consistent with the Masquelet technique. The successful reconstruction of a 12-cm radial defect using an NVFG in this setting underscores that the biological environment, infection control, and mechanical stability are more decisive factors for union than graft vascularity alone.

Adequate soft-tissue coverage is a critical determinant of success in complex open forearm injuries. In situations where microsurgical reconstruction is not feasible, pedicled groin flaps provide a reliable and time-tested option for covering extensive upper-extremity defects. Despite drawbacks such as temporary immobilization and the need for a secondary division procedure, this technique remains highly valuable in resource-limited environments because of its reliability, simplicity, and robust vascular supply [9].

In this case, groin flap coverage, combined with staged debridement, external and intramedullary stabilization, and temporizing biological reconstruction, created a stable environment for definitive bone grafting. This staged approach enabled successful graft incorporation, complete soft-tissue healing, and restoration of limb stability despite delayed evacuation, limited resources, and prolonged treatment intervals [10].

Although limb salvage was ultimately successful, persistent wrist drop reflects the severity of the initial injury and highlights the frequent need for secondary reconstructive procedures in blast-related upper-extremity trauma. Overall, this case demonstrates that staged, biology-driven reconstruction using an NVFG and pedicled groin flap coverage can achieve reliable limb salvage even in the absence of advanced microsurgical capabilities.

## Conclusions

Reconstruction of large segmental radial bone defects with extensive soft-tissue loss remains a major surgical challenge, particularly in conflict-affected and resource-limited settings. This case demonstrates that a staged limb-salvage strategy combining meticulous debridement, infection control, NVFG, and pedicled groin flap coverage can achieve reliable bone union, stable soft-tissue reconstruction, and satisfactory functional outcomes, even in the absence of microsurgical capabilities. This approach provides durable soft-tissue coverage, supports the biological conditions necessary for graft incorporation, and restores structural integrity, highlighting the importance of a well-prepared reconstructive environment before definitive surgery. However, residual functional deficits, particularly those related to nerve and tendon injuries, may persist and require secondary procedures and rehabilitation. Overall, this case supports NVFG as a practical and effective alternative when combined with staged reconstruction for complex upper-extremity injuries when advanced reconstructive options are not available.
